# The Role of Methyl-(Z)-11-tetradecenoate Acid from the Bacterial Membrane Lipid Composition in *Escherichia coli* Antibiotic Resistance

**DOI:** 10.1155/2022/6028045

**Published:** 2022-06-13

**Authors:** Alexandru O. Doma, Romeo T. Cristina, Florin Muselin, Eugenia Dumitrescu, János Dégi, Kálman Imre, Marius Boldea, Daliborca C. Vlad, Roxana Popescu, Adinela Cimporescu, Dana C. Bratu

**Affiliations:** ^1^Banat's University of Agricultural Sciences and Veterinary Medicine Timisoara, Calea Aradului 119, 300645 Timisoara, Romania; ^2^University of Medicine and Pharmacy Timisoara, Piata Eftimie Murgu, 2, 300041 Timisoara, Romania

## Abstract

**Background:**

The bacterial membrane plays a critical role in the survival of bacteria and the effectiveness of antimicrobial peptides in protecting the host. The lipid constituents of the bacterial membrane are not evenly distributed, and they could be affected by clustering anionic lipids with cationic peptides with multiple positive charges. That could be harmful to bacteria because it prevents lipids from interacting with other molecular components of the cell membrane, disrupts existing natural domains, or creates phase boundary defects between the clustered lipids and the bulk of the membrane. This preliminary quantitative study is aimed at assembling a correlation between antibiotic resistance and bacterial lipid composition in *E. coli*, based on the function and arrangement of the bilipid coating of the bacterial cell, intimately associated with the path of antimicrobials through membranes.

**Methods:**

Fifteen multiresistant *E. coli* samples are collected from swine with enterocolitis tested for resistance levels using the disc diffusimetric method (Kirby-Bauer disc diffusion). Pathogen identification completed using the API 20E multitest system revealed the *E. coli* presence in 11 samples. In these samples, bacterial membrane detection of fatty acid methyl esters (FAME) operating a 240 MS Ion Trap (Varian) GC/MS (Agilent Technologies, Santa Clara, CA, USA) was performed, using the MIDI Sherlock recognition software model.

**Results:**

Interpreting the descriptive statistical method, the correlation matrix, and regression curves and after ANOVA analysis, we ascertained that the studied *E. coli* population statistically confirmed different degrees of resistance in most of the samples analyzed in this test.

**Conclusions:**

In one case, the methyl-(Z)-11-tetradecenoate acid was observed to have a relationship with the susceptibility evaluation by using the disc diffusimetric method, which has revealed the lowest rate of antimicrobial resistance, so it has importance in further resistance evaluation studies.

## 1. Introduction

Antimicrobial resistance (AMR) is the capability of microorganisms to adapt to antimicrobials, mainly antibiotics. Exaggerated and inappropriate use of antimicrobials and inefficient infection management approaches resulted in AMR being a serious global public health menace. Databases and supervision systems from both human and veterinary sectors are becoming increasingly abundant in data since. As of today, resistance is registered for almost all antibiotics [1, 2].

Though antimicrobials have revised medical practices today, this benefit is a distinct threat due to the intense or inappropriate antibiotic administration. The irresponsible use of antibiotics has expanded the occurrence and spread of multidrug-resistant bacteria driving the optimization of veterinary antimicrobial treatment as a crucial concern. Along with antibiotics employed in human therapy, their use to cure infections or for prophylactic treatments in animals has led to pressure on the emergence and quick spread of resistant bacterial strains [3]. Recent studies described that faulty antimicrobial use in slaughterhouse animals could influence the health of farmworkers and employees from meat processing units or the final consumer health. Why is resistance spreading so quickly, and what are their intimate/specific resistance mechanisms? They are not fully established yet [3].

Animals can operate as intermediates, pools, and disseminators of resistant bacterial strains or genes. The alarming augment in reports about multidrug-resistant (MDR) bacteria, combined with the decline in new drug approvals, is indisputable that this reduces the supply of clinical treatment options [4–7]. In these conditions, attempts to manage bacterial infections remain challenging, spoiling the efficacy of infectious diseases' treatments. As a result, the need for renewed infection control strategies is becoming crucial [6, 7].

The bacterial membrane plays a critical role in the survival of bacteria and the effectiveness of antimicrobial peptides in protecting the host, and Gram-negative bacteria's outer membrane has an additional layer of protection [8, 9].

The outer leaflet of these bacteria's outer membrane is composed mainly of lipopolysaccharide (LPS), the major component of the outer membrane of Gram-negative bacteria localized in the outer layer of the membrane. Phosphatidylglycerol (PG), phosphatidylethanolamine (PE), and cardiolipin are the main lipid elements of the inner monolayer of Gram-negative bacteria's outer membrane, as well as both monolayers of their cell membranes (CL). The bacterial species vary considerably in the proportions of these three lipids [10–13]. Also, certain bacterial species include significant quantities of glycosyl-diglycerides and a mixture of other minor lipid components [10–13]. Numerous antimicrobial agents exert their activity by interfering with the synthesis of mature LPS at different stages. This action enhances the susceptibility of these bacteria to destruction by making the outer membrane more permeable, driving this additional protective layer inefficient. Surprisingly, there are some examples where the antimicrobial agents avert solutes from passing through the outer membrane of Gram-negative bacteria, resulting in their lethality [14]. The lipid components of biological membranes are not distributed equally throughout the membrane but supplemented in specific domains, and this dispersal is altered by clustering anionic lipids with cationic peptides with multiple positive charges [15–17]. This could be harmful to bacteria, since it prevents lipids from interacting with other molecular components of the cell membrane, disrupts existing natural domains, or creates phase boundary defects between the clustered lipids and the bulk of the membrane [18].

The lipid composition of bacterial membranes differs significantly between species [19]. Among these unknowns, the fatty acid function in Gram-negative bacteria membrane is not completely understood yet, though the studies demonstrate that these lipid configurations may impact the penetration or binding of antimicrobials. For example, tetracycline [20, 21] is dependent on membrane permeability, penicillin, and quinolones on high lipid content [22–24]. Furthermore, the viability of Gram-negative bacteria is associated with the integrity of the cell membrane, and lipids could play a role in their structure and function due to the quantitative differences in fatty acids. For this reason, researchers have developed comprehensive investigations in this direction [25, 26].

Scientific reports have presented diverse techniques for differentiating fatty acids with a length of 9-20 carbons. Fatty acids with a short chain were found in Gram-negative bacteria, and those with a branched chain were found in Gram-positive bacteria. These techniques help identify and classify them. The microorganisms are isolated and cultured on selective media, in temperature and humidity optimal conditions, and subjected to tests to highlight fatty acids in their bacterial membrane [27].

The present study tries to assemble an initial qualitative correlation between antibiotic resistance and bacterial lipid composition in *E. coli*. The function and structure of the bilipid coating of the bacterial cell are intimately related to the passage of antimicrobials through membranes. To reach the proposed goals, this was followed:

- Simple identification of the pathogen using the API 20E multitest system
- Setting the level of resistance by the disc diffusimetric method
- Ascertaining the fatty acids in the bacterial membrane by gas chromatography detection of fatty acid methyl esters (GC-FAME)
- Assembling all possible statistical correlations

To our knowledge, this is a prime initial study made in Romania on *Enterobacteriaceae*.

## 2. Materials and Methods

All procedures used in this study by providing clinical samples and written informed consent have the approval of the Ethical Committee of USAMVB Timișoara (Ethical Committee Approval no. 164/05.12.2019).

### 2.1. Location and Sample Collection for AST

For this research, large capacity swine exploiting units from Arad County, Western Romania, have been included, where clinical cases were diverse, and the identified incidence of colibacillary infections was high. The examination was completed on biological material from pure *E. coli* cultures (of maximum 20 mg), gathered directly from the fresh intestinal contents of swine.

### 2.2. Bacterial Isolation and Microbial Testing

During a two-year period, a total of 15 *E. coli* strains from the level of the small intestine of pigs having acute diarrheal diseases were recovered. The bacteria isolation was made, following the standard protocol within the bacterial determinations. MacConkey (MAC; Merck, Darmstadt, Germany) agar specific was used to develop *E. coli* strains. The lactose-fermenting (pink) *E. coli* colonies were subsequently selected and cultured on triple sugar iron (TSI; Oxoid, Basingstoke, UK) slopes and tryptone soy agar (TSA, Oxoid, Basingstoke, UK). In addition, the presumptive *E. coli* colonies were Gram-stained and cultured on TSA agar to be identified based on their biochemical properties with the API 20E typing system (BioMérieux, Marcy L'Etoile, France).

The ATCC 25922 *E. coli* was used as a control strain, with whole bacterial strains maintained on nutrient agar. From the collected samples, the bacterial resistance of the 15 isolates was initially tested. The susceptibility was tested through the Kirby-Bauer disc diffusion standardized technique, completed by measuring the diameter of the growth inhibition zone. The strains were classified as resistant or sensitive to the drug, according to the current interpretation standards presented in the Clinical Laboratory Standards Institute (CLSI), Performance Standards for Antimicrobial Disc Susceptibility Tests [28–30].

### 2.3. Analytical Profile Index (API)

For the biochemical characterization of *E. coli* was used the API 20E kit (BioMérieux, Marcy L'Etoile, France). Twenty plastic tubes (cups) containing dehydrated reagents comprise the test strip. After 16-24 hours of culture with a bacterial solution, the bacterial metabolites change the color of the domes, allowing the identification of different bacterial species. The six reagents included in this test are glucose (GLU), D-mannitol (MAN), inositol (INO), D-sorbitol (SOR), D-sucrose (SAC), and amygdalin (AMY), and each contains a pH indicator. We have used these six reagents since the *Enterobacteriaceae* have a common biochemical metabolic pathway known as mixed acid fermentation, specific for the metabolism and breakdown of sugars, such as glucose, and the production of various acidic products that can cause the reagents to change color due to the acid-base indicator [4, 31].

Compared to the classical methods, the use of multitest identification systems has benefits: receiving rapid results, in some circumstances even within 5 hours; uniformity of results; precision/safety of results; simplicity of work procedures; and lowest consumption of materials and culture media [4, 31]. The final evaluation involved the registration of positive (+) or negative (-) results and the species identification completed by consulting the Index supplied by the manufacturer. Of the 15 samples investigated, in 11 cases, *Escherichia coli* were present, and consecutive confirmation of the results was identified in resistant and sensitive species to antibiotics investigated. Specimens were tested to determine the fatty acids in their bacterial membranes, using the GC-MS technique with numerous methyl ester compounds identified.

### 2.4. GC-MS Methodology and the Study of Bacterial Membrane Fatty Acids

Short-chain fatty acids typically identify the anaerobic bacteria, especially the acids having between 9 and 20 carbon bonds [27]. With gas chromatography, the fatty acid methyl esters (GC-FAME) could be determined. The richness of information contained in these compounds was estimated, considering the presence/absence of each acid and the acid fluctuation existing associated with the development of antimicrobial resistance [27, 32].

Bacterial growth conditions can influence the accumulation of fatty acids in membranes. Quantitative and qualitative fluctuations were reported, depending on the incubation temperature and the growth media used. For these variables minimization, using a TSA medium (Trypticase Soy Agar, Sigma-Aldrich, Darmstadt, Germany) incubated at 28°C or a TSBA (Trypticase Soy Blood Agar, Sigma-Aldrich, Darmstadt, Germany) at 35°C for 24 hours is recommended.

The mass spectrometry (MS) used a 240 MS Ion Trap (Varian, Santa Clara, CA, USA) mass spectrometer, with the ionization conditions set as follows: ion trap temperature at 170°C and the transfer line temperature at 230°C using helium as mobile phase. The EI mode at 70 eV was used for ionization, and the compounds' nominal mass recording used the full spectra. Each standard was injected separately to check if the fragmentation data matched the database information. Fragmentation patterns were extracted from the identified peaks and referred to the spectral database (Wiley Registry/NIST 2020 Library/12^th^ Edition) for the confirmation of the identity of the separated compounds.

Chromatography was performed on 450 GC Varian (Varian, Santa Clara, CA, USA) using hydrogen as carrier gas at a constant flow rate of 1.5 mL/minute and a linear velocity of 50.1 cm/sec. For qualitative analysis, a column BR-5ms (30 m × 0.25 mmID, 0.25 *μ*m film) (Brucker Daltonics, MA, USA) was maintained at a temperature of 100°C, 1 min, raised to 300°C with a rate of 10°C/min, held for 2 min. The set injection pool's temperature was 230°C. Samples were injected at a volume of 1 *μ*L using a split ratio for 5 min, followed by splitless mode for 10 min [27, 33, 34].

As the GC-FAME analysis is used to identify bacterial species, the fatty acid profile is a certain indication of these species. This is why we designed this study observing the fatty acid profile composition of *E. coli* in strains that are and are not resistant to the same antibiotic considering that any registered modifications are associated with the resistance presence/absence [27, 33, 34].

For these determinations, four preliminary operations were made as shown in Table 1.

## 3. Results

The investigation of swine samples, confirming the failure of antibiotic therapy, started with the pathogen's identification using the API methodology (API kit 20E, Biomerieux, Marcy L'Etoile, France). The disc diffusimetric method followed the CLSI 2018 instructions to test bacterial susceptibility [28, 29].

Table 2 presents the interpretation of antibiotic susceptibility.

**Figure 1:**
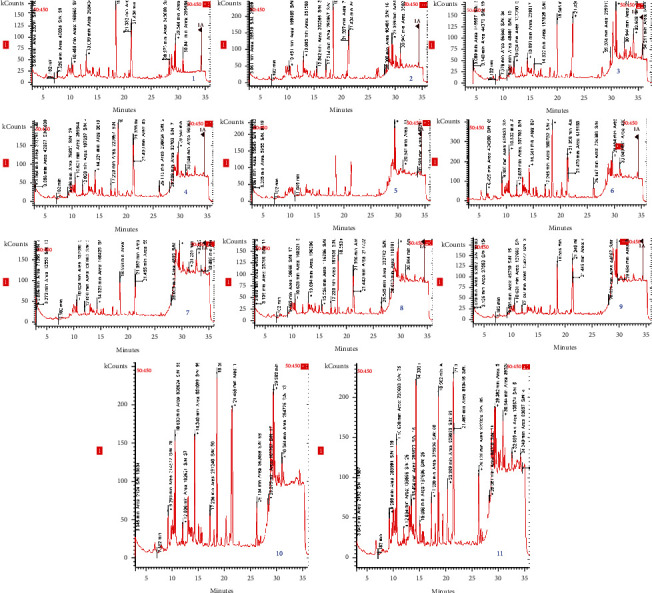
The chromatograms of the identified fatty acids.

Gas chromatography identified fatty acids extracted with the help of methanol. The obtained chromatograms are presented in Figure 1.

Results of the 11 chromatograms (collection dials) qualification and the highlighting of the fatty acids in the bacterial membrane of *E. coli* depending on the retention time are presented in Table 3.

### 3.1. Statistic Assays and Correlation

#### 3.1.1. Descriptive Statistics

**Figure 2:**
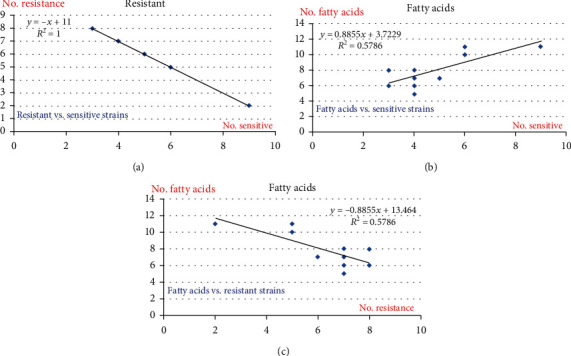
The descriptive statistic correlation between (a) the fatty acids vs. sensitive strains and (c) fatty acids vs. resistant strain presence/absence in the bacterial membrane, where a high significance correlation was registered for (b, c).

Analyzing after the descriptive statistic correlation between matrix and regression curves had shown that the *E. coli* population studied statistically confirmed different degrees of resistance in most of the samples analyzed (Table 4 and Figure 2).

Figure 2 presents the descriptive statistic correlation between (a) fatty acids vs. sensitive strains and, respectively, (c) fatty acids vs. resistant strain presence/absence in the bacterial membrane, where a high significance correlation has been registered for b and c.

#### 3.1.2. ANOVA

In addition, to ensure the accuracy of the results, they were statistically analyzed also by the bidirectional ANOVA method, using the GraphPad Prism 6.0 program for Windows (GraphPad Software, San Diego, USA). Statistical values were expressed as the mean ± SEM (standard error), where ∗ means 0.01 ≤ *p* < 0.05, significant; ∗∗ means 0.001 ≤ *p* < 0.01, very significant; and ∗∗∗ means p < 0.001, extremely significant (Table 5).

The obtained statistical values were highly significant, and the matrix of statistical correlation for fatty acids vs. sensitive strains' and fatty acids vs. resistant strains' presence/absence in the bacterial membrane confirmed the descriptive statistic correlation made for b and c.

## 4. Discussion

The last decade had shown that there is an urgent need for new antibiotics against microbial infections. The incidence of microbial resistance to traditional antibiotics has advanced continuously [16, 35], and in this case, the bacterial cell membrane is one of the possible targets of new antibiotics that may be effective against resistant bacteria [36, 37].

A recent ample analysis highlighted the antimicrobial peptides' characteristics that interact with bacterial membranes [38, 39]. The assessment of the portion of the fatty acids, compared to the sensitivity rate of the antibiotics used in present study, was higher in phenotype S, compared to phenotype R, a fact also found by other researchers. They identified significant differences in terms of the quantitative fatty acid composition of sensitive versus resistant strains [25, 36, 37].

Gram-positive bacteria membranes are fundamentally different in their molecular composition and morphology from Gram-negative bacteria membranes. Gram-negative bacteria are encased in two membranes: the cytoplasmic and outer membranes. The outer monolayer of the membrane is primarily composed of LPS, a lipid species found only in Gram-negative bacteria [40, 41].

The most notable fact in the present research was that it confirmed the proportional reductions in acids in the resistant strains, compared to the sensitive strains of *E. coli*. It is known that antibiotics from the *β*-lactam category are targeting penicillin-binding membrane proteins in the cell wall. They are hydrophilic and cross the outer membrane with the help of porins through a process mediated by the physicochemical properties of the molecule, and quinolones focus on cytoplasmic enzymes (DNA gyrase and topoisomerase IV), so these substances must cross the cell wall to be effective [3, 25, 42]. For these antimicrobials, the resistance is correlated with the outer membrane diffusion channel descent through the porins and oversizing of the efflux pumps [23, 24].

Like other authors, our results fall in that fatty acids can influence the permeability of antibiotic molecules by acting directly on transmembrane proteins or collectively by impacting the fluidity, size of the bilipidic layer, and the shape they can give to the membrane [3, 26, 42].

The activity of the used antibiotics was not directly correlated to the metabolism of fatty acids but may indirectly affect the membrane permeability, contributing to the development of antibiotic resistance [43, 44].

It is acknowledged that, at high levels of saturated fatty acids, the fluidity of the membrane decreases. This decrease has been reported by authors in the case of exposure to subinhibitory concentrations of quinolones and cephalosporins in *E. coli* [45–47].

Variation in cyclopropane fatty acids is a response to antibiotic exposure of tetracycline-resistant strains of *E. coli* so that the amount of cyclopropane fatty acids decreases and the concentration of unsaturated fatty acids increases compared to susceptible phenotypes [22]. Although changes in the composition of fatty acids in the bacterial membrane reflect the differences in phenotype in terms of antimicrobial susceptibility, their interactions can be hard to interpret, and relatively, little is known yet about these interferences [9].

In this respect, even though this study is an initial and a qualitative investigation, it brings new data and can generate the following in what means multifaceted analysis of qualitative interrelations between the bacterial membrane's fatty acid presence/absence vs. sensitive/resistant *E. coli* strains.

## 5. Conclusions

The presence of methyl-(Z)-11-tetradecenoate acid ascertained was in positive connection (where *p* < 0.01) with the susceptibility of *E coli*; this fatty acid was not found in the resistant *E. coli* strains identified. This allows us to affirm that the bacterial fatty acid amount and composition could reflect the differences in antimicrobial susceptibility.

In order to elucidate all interactions, more research would be opportune to continue this initial study, by observing the fatty acids' structure variation in the bacterial membranes and the link with the antibiotics' efficiency, with valuable outcomes in the combat against antibioresistance.

## Figures and Tables

**Table 1:** Sample preparation and reagents used.

Phase	Substances used
Saponification	Reagent 1: (i) 45 g sodium hydroxide (ii) 150 mL methanol (iii) 150 mL distilled water
Methylation	Reagent 2: (i) 325 mL clorhidric acid 6.0 N (ii) 275 mL methanol
Extraction	Reagent 3: (i) 200 mL hexane (ii) 200 mL methyl tert-butyl-ether (MTBE)
Rinse	Reagent 4: (i) 10.8 g sodium hydroxide (ii) 900 mL distilled water

**Table 2:** The antibiotic susceptibility results according to CLSI 2018 [29].

Crt. No.	Sample no./antibiotic tested	1	2	3	4	5	6	7	8	9	10	11
1.	Amoxicillin	R	R	R	R	R	R	R	R	R	R	R
2.	Gentamycin	S	S	S	S	S	S	S	S	S	S	S
3.	Florfenicol	R	S	R	S	R	S	R	R	R	R	R
4.	Oxacillin	S	R	R	R	R	S	S	S	S	S	S
5.	Cephalothin	R	R	R	R	R	R	R	R	R	R	R
6.	Spectinomycin	R	R	R	R	R	S	R	R	R	R	S
7.	Norfloxacin	R	S	S	S	S	S	R	R	S	R	S
8.	Tiamulin	S	S	S	S	S	S	S	S	R	S	S
9.	Ciprofloxacin	S	S	R	R	R	S	R	S	R	S	R
10.	Penicillin	R	S	R	R	R	S	S	R	R	R	S
11.	Tetracycline	R	R	R	R	R	S	S	R	S	R	R

Note: R: resistant; S: sensitive.

**Table 3:** Quantification of results and highlighting of fatty acids in the bacterial membrane.

Retention time (min.)	Identified compound	No. of collection dial (chromatogram)										
		1	2	3	4	5	6	7	8	9	10	11
8.2	Decanoic acid (methyl ester)	−	+	−	−	+	−	−	−	−	−	−
10.3	4-Octadecenal	−	−	−	−	−	+	−	−	−	−	−
10.6	Methyl-10-methyl undecanoate	−	+	+	+	+	+	+	+	+	+	+
13.8	Methyl-(Z)-11-tetradecenoate	−	−	−	−	−	+	−	−	−	−	−
14.3	Methyl-iso-myristate	+	+	+	+	+	+	+	+	+	−	+
15.7	Methyl-(Z)-11-tetradecenoate	−	−	−	−	−	−	−	−	−	+	+
17.2	I-propyl-tetradecanoate	+	+	+	+	+	+	+	+	+	+	+
20.1	Methyl-palmitoleate	+	+	+	+	+	−	−	−	+	+	−
20.2	Methyl-hexadec-9-enoate	−	+	−	−	−	+	+	−	−	−	+
21.3	Methyl-14-methyl-pentadecanoate	−	−	−	+	−	+	+	+	−	−	+
21.4	Methyl-3-(3.5-di-tert-butil-4-hidroxy-phenyl) propionate	+	−	−	−	−	−	−	−	−	−	−
26.0	Methyl-8-heptadecenoate	−	+	−	−	−	−	−	−	+	−	−
26.1	Methyl-9,10-metylene-hexadecanoate	−	−	+	−	−	+	+	−	−	+	+
26.1	Cis-10-heptadecenoic-acid (methyl ester)	+	−	−	+	+	−	−	−	−	−	−
29.0	Methyl-(10E)-10-octadecenoate	−	−	−	+	−	−	−	−	−	−	−
29.0	Methyl-11-octadecenoate	+	−	−	−	+	+	−	−	−	−	−
29.0	Methyl-trans-8-octadecenoate	−	+	−	−	−	−	−	−	−	−	−
29.1	Methyl-13-octadecenoate	−	−	−	−	−	−	−	−	−	−	+
29.5	Methyl-16-methyl-heptadecanoate	−	−	+	+	+	−	+	−	+	−	−
29.5	Methyl-14-methyl-heptadecanoate	−	+	−	−	−	+	−	−	−	+	+
30.4	Decanoic acid (decyl ester)	+	+	−	−	−	+	−	+	−	+	+
31.2	Methyl-dihydrosterculate	−	−	−	−	−	−	−	−	−	+	−
31.2	Methyl-9,10-metilene-octadecanoate	−	−	−	−	−	−	−	−	−	−	+

**(a):** 

Sample no.	Sensitive no.	Resistant no.	Fatty acid no.
1	4	7	7
2	6	5	10
3	3	8	6
4	4	7	8
5	3	8	8
6	9	2	11
7	5	6	7
8	4	7	5
9	4	7	6
10	4	7	8
11	6	5	11
Mean	4.727272727	6.272727273	7.909090909
Standard error	0.523813101	0.523813101	0.609836721
Median	4	7	8
Mode	4	7	8
Standard deviation	1.737291518	1.737291518	2.022599587
Sample variance	3.018181818	3.018181818	4.090909091
Kurtosis	3.033156723	3.033156723	-0.866962963
Skewness	1.629735293	-1.629735293	0.416039579
Range	6	6	6
Minimum	3	2	5
Maximum	9	8	11
Sum	52	69	87
Count	11	11	11
Confidence level (95.0%)	1.167128323	1.167128323	1.358800892

**(b):** 

Sensitive		Resistant		Fatty acids	
Lower	Higher	Lower	Higher	Lower	Higher
4.7273-1.1671	17273 + 1.1671	6.2727 − 1.1671	6.2727 + 1.1671	7.9091 − 1.3588	7.9091 + 1.3588
3.560144	5.894401	5.105599	7.439856	6.55029	9.267892

**(a):** 

ANOVA	SS	dF	MS	*F* (DFn.DFd)	*p* value/significance
Treatment (between columns)	55.70	2	27.85	*F* (2.30) = 8.25	*p* = 0.0014
Residual (within columns)	101.30	30	3.37		
Total	157	32	31.22		
ANOVA summary					
*F*					8.25
*p* value					0.0014
*p* value summary					∗∗
Are differences among means statistically significant? (*p* < 0.05)					Yes
*R* square					0.3548

**(b):** 

Parameter	Fatty acids vs. sensitive	Fatty acids vs. resistant
Pearson *r*		
*r*	0.7606	-0.7606
95% confidence interval	0.2955 to 0.9343	-0.9343 to -0.2955
*R* square	0.5786	0.5786
*p* value		
*p* (two-tailed)	0.0066	0.0066
*p* value summary	∗∗	∗∗
Significant? (alpha = 0.05)	Yes	Yes
Number of *XY* pairs	11	11

**(c):** 

Summary				
Groups	Count	Sum	Average	Variance
Sensitive	11	52	4.727272727	3.018182
Resistant	11	69	6.272727273	3.018182
Fatty acids	11	87	7.909090909	4.090909

**Table:** (d) The matrix of statistical correlation

Category	Sensitive	Resistant	Fatty acids
Sensitive	1	-1	0.760627515
Resistant	-1	1	-0.760627515
Fatty acids	0.760627515	-0.760627515	1

## Data Availability

All data is contained within the article.
